# Patients With Severe Obesity Are Made Eligible for Complex Abdominal Wall Repair After Preoptimization With GLP‐1 Agonists: Results of a Bicentric Pilot Study

**DOI:** 10.1002/wjs.12547

**Published:** 2025-03-15

**Authors:** Benoit Romain, Vincent Pfirsch, Simone Manfredelli, Thomas Leroi, Fadi Salman, Ouidad Sami, Diane Westerfeld‐Ruillier, Séverine Ledoux, David Moszkowicz

**Affiliations:** ^1^ Service de Chirurgie Générale et Digestive Hôpital de Hautepierre Hôpitaux Universitaires de Strasbourg Strasbourg France; ^2^ Materials, Multiscale and Biomechanics Team Département de Mécanique ICUBE Laboratory Strasbourg France; ^3^ Etablissement SMR des Trois‐Epis Trois Epis France; ^4^ Service de Chirurgie Digestive AP‐HP Hôpital Louis Mourier DMU ESPRIT‐GHU AP‐HP Nord‐Université Paris Cité Colombes France; ^5^ Explorations fonctionnelles Centre Intégré Nord Francilien de l’Obésité AP‐HP.Nord – Université Paris Cité Colombes France; ^6^ UGECAM Alsace Illkirch‐Graffenstaden France

**Keywords:** GLP‐1 agonists, incisional hernia repair, obesity, prehabilitation, weight loss

## Abstract

**Background:**

Incisional hernia repairs (IHRs) are not recommended in patients with severe obesity (BMI ≥ 35 kg/m^2^). Weight loss is challenging, but new medications, such as glucagon‐like peptide‐1 receptor agonists (GLP‐1 agonists), have recently attracted increased attention for their potential weight loss advantages. The aim was to analyze the preliminary results about the safety and weight loss efficiency of the use of GLP‐1 agonists in the context of prehabilitation prior to complex IHR.

**Methods:**

All patients planned for IHR with a BMI ≥ 35 kg/m^2^ and treated with preoperative GLP‐1 agonists were included in the experimental group and compared with a comparable historical surgical cohort treated with a conventional tailored nutritional preoperative management. Weight loss in the experimental group and perioperative and postoperative outcomes were compared between the two groups. The success rate of GLP1 agonists was defined as a weight loss that enables the patient to fall within the recommended limits of a BMI ≤ 35 kg/m^2^ before an IHR.

**Results:**

Fifty‐two patients in the control group were compared to 24 with GLP‐1 agonists. The distribution of GLP‐1 agonists was as follows: semaglutide (*n* = 12; 50%), dulaglutide (*n* = 7; 29.2%), and liraglutide (*n* = 5; 20.8%). The mean initial BMI was 40.1 ± 3.6 kg/m^2^ kg/m^2^. The average percentage of weight loss was 11.3 ± 7.4% with GLP‐1 agonists (maximum weight loss was observed with semaglutide 2.4 mg/wk). The success rate of GLP1 agonists (defined as BMI ≤ 35 kg/m^2^ before IHR) was reached for 15/24 patients (62.5%). Postoperative total complication rate was lower in the group with GLP‐1 agonists (59.6% in the control group vs. 45.8% in GLP‐1 and *p* = 0.2).

**Conclusion:**

This study demonstrated the efficacy of GLP‐1 agonists in the optimization of patients with obesity, allowing two thirds of the patients to benefit from IHR, with a tendency for lower morbidity.

**Trial Registration:** CPP Mediterranee, n° 21.00430.000004.

## Graphical abstract

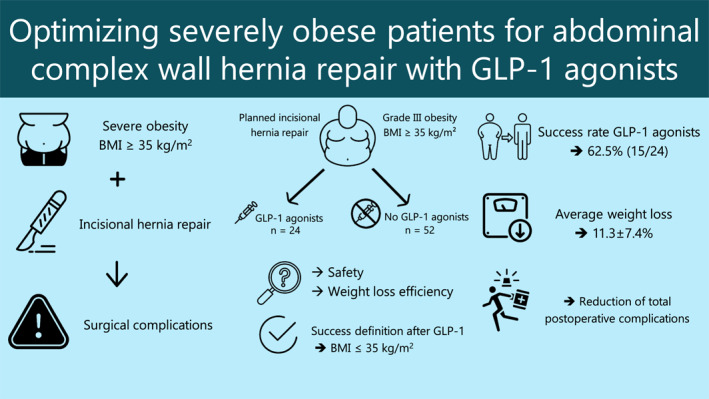

## 1 Introduction

Complex abdominal wall surgery causes frequent postoperative morbidity especially in patients with comorbidities. Prehabilitation is a preparation program prior to surgery in order to limit postoperative complications and improve recovery by preparing patients in advance of major surgery [1]. Obesity is a known risk factor for complications and recurrence after incisional hernia repair (IHR) [2]. The prevalence of obesity has dramatically increased worldwide, particularly among patients indicated for abdominal wall surgery [3]. To optimize outcomes and limit postoperative risks, guidelines have adopted BMI cutoffs for surgery requiring patients to attempt to pursue weight loss [4]. However, weight loss is difficult to achieve given a combination of factors, including societal and biological counteracting factors. Preoperative weight loss is not always possible due to symptoms or emergency management, that is responsible of early recurrence and increased risk of loss of abdominal wall domain.

Therefore, there are currently limited options for patients with obesity pursuing weight loss to undergo IHR. Diet, lifestyle modification, and exercise rarely make it possible to achieve weight loss objectives. Incisional hernia repair (IHR) is now one of the indications for bariatric surgery in patients with obesity in France [5], but it is not always possible in such complex patients. Thus, pharmacological options have recently expanded.

Glucagon‐like peptide‐1 (GLP‐1) agonists were initially designed as a medication for diabetes [6], but these agents are also efficient for weight loss in patients with obesity [7]. GLP‐1 is an incretin, the role of which is the stimulation of insulin release and also promoting satiety by slowing gastric emptying and via a central effect [8]. There are few studies evaluating the potential effects of GLP‐1 receptor agonists as adjuvant treatment for several surgical interventions. Some recent articles have describe significant weight loss in patients after orthotopic liver transplant, potentially optimizing outcomes and survival [9, 10]. Similarly, GLP‐1 receptor agonists may have modest glycemic and weight benefits in patients who undergo renal transplantation [10]. Semaglutide therapy has shown efficacy as an adjunct to maintain postbariatric surgery weight loss [11]. Semaglutide use during total knee arthroplasty has been suggested to decrease the risk of sepsis, prosthetic joint infections, and readmissions [11]. By significantly reducing excess weight, the use of GLP‐1 analogs could play an important role in the prehabilitation of obese patients undergoing incisional hernia surgery in order to reduce postoperative morbidity. Thanks to the reimbursement of GLP‐1 agonists using the French health insurance system for a certain period of time, the use of such treatments has become possible. The aim of this article was to analyze the preliminary results about the safety and weight loss efficiency of the use of GLP‐1 agonists in the context of prehabilitation prior to complex abdominal wall surgery.

## 2 Methods

### 2.1 Study Design

Patients operated on abdominal wall surgery in 2 tertiary university surgical departments with obesity and complex abdominal wall surgery expertise (CAWR) were included in a database. According to Slater et al, the definition of ‘‘complex abdominal wall hernia’’ derived from 4 criteria: defect size and location, patient history and risk factors, contamination and soft tissue condition, and clinical scenario [12]. Severe obesity was definied as a BMI ≥ 35 kg/m^2^ [13]. Two groups were set up before and after the introduction of GLP‐1 receptor agonists. Group 1 corresponded to consecutive patients with a BMI ≥ 35 kg/m^2^ before the GLP‐1 agonist era from January 2019 to June 2021, whereas Group 2 corresponded to patients with GLP‐1 agonists from January 2021 to May 2024. Group 1 patients were taken from a historical series of patients who had undergone IHR but who had not benefited from the current prehabilitation recommendations. All patients, irrespective of the group that they were in, benefited of a tailored nutritional management with the help of nutritionists and dieticians. They assessed the nutritional status of each patient, taking into account their usual food intake. They gave standardized advices on how to achieve a balanced diet emphasizing physical activity. Depending on the situation, several follow‐up consultations could be organized. Preoperative and intraoperative patient data were collected using the medical chart review. Data collection was validated by the national ethics committee (CPP Méditerranée, n° 21.00430.000004).

### 2.2 Data Collected

Demographic, preoperative, and operative characteristics were recorded based on clinical files. For the experimental group, we assessed the BMI between the initial consultation and the date of the operation. The BMI for the control group was that on the date of the operation. The other data collected were age, sex, GLP‐1 agonist types, safety and tolerance of the GLP‐1 agonist used, previous comorbidities and risk factors (smoking, obesity, and diabetes), VHWG (Ventral Hernia Working Group) score, and incisional hernia size according to EHS guidelines [14]. The success rate of GLP1 agonists was defined as a weight loss that enables the patient to fall within the recommended limits of a BMI ≤ 35 kg/m^2^ before an incisional hernia repair (IHR).

In France, during a limited period of temporary authorization for research use, patients could benefit from the use of Wegovy (semaglutide, 2.4 mg/wk) that was only been assured. This allowed us to include only a limited number of patients. At present, the drug has obtained marketing authorization for patients with a BMI greater than or equal to 35 kg/m^2^ and aged under 65, but it is not reimbursed using the French health insurance system. As a result, it is only available to a limited number of patients due to the cost of the treatment (250 euros/month). Prescriptions were made by nutritionists at our centers. The use of GLP1 agonists in this indication is in its early stages and has not been standardized. As a result, the use of agonists is not homogeneous and doses are not necessarily maximal. GLP‐1 agonists could also be prescribed when there was an ineffective weight loss or weight regain, once the dietary, psychological, and physical activity parameters had been controlled and in the absence of any contraindication or intolerance.

The efficacy of different GLP‐1 analogs was analyzed: semaglutide (Ozempic and Wegovy), dulaglutide (Trulicity), and liraglutide (Saxenda and Victoza). Among adults with overweight or obesity without diabetes, once‐weekly subcutaneous semaglutide compared with once‐daily subcutaneous liraglutide or dulaglutide, added to counseling for diet and physical activity, resulted in significantly greater weight loss [15, 16]. This is why we studied the higher dosage of semaglutide compared with other GLP‐1 agonists and Wegovy at 2.4 mg/week is the highest dosage of semaglutide.

The surgical procedure was not detailed because it was not the focus of this present study. Surgery was performed using laparotomy with the placement of a mesh in a retromuscular position. Patients with a hernia width ≥ 10 cm received botulinum toxin injections for optimization 4–6 weeks before surgery into the lateral abdominal muscles. Patients with loss of domain underwent prehabilitation with progressive pneumoperitoneum to minimize the risk of postoperative abdominal compartment syndrome. Anterior (Ramirez) or posterior (Transverse Abdominis Release) component separation techniques were used, if necessary.

Wounds events reporting were standardized according to recommendations [17]. Surgical site infection (SSI) was defined by superficial and deep infections [18]. Surgical site occurrence (SSO) was defined by cellulitis, SSI, seroma, hematoma, enteral fistula, infected mesh, and wound disunion. Complications were graded according to the Dindo–Clavien classification [19]. A complication of grade III or above was considered serious and grade II or below was considered minor. Only the most serious complication was taken into account. Postoperative follow‐up was deliberately limited to the immediate postoperative period (day 30) in order to collect early postoperative morbidity.

### 2.3 Statistical Analysis

Qualitative variables were described as percentages and compared with a chi‐squared test or Fisher's exact test as appropriate. Quantitative variables were described with their mean and standard deviation and/or as a median and interquartile range. Student's *t* test was used to compare means of continuous variables if they were normal and the Mann–Whitney–Wilcoxon test was used if they were not. The threshold of significance was set at *p* < 0.05.

## 3 Results

### 3.1 Demographic Data

**FIGURE 1:**
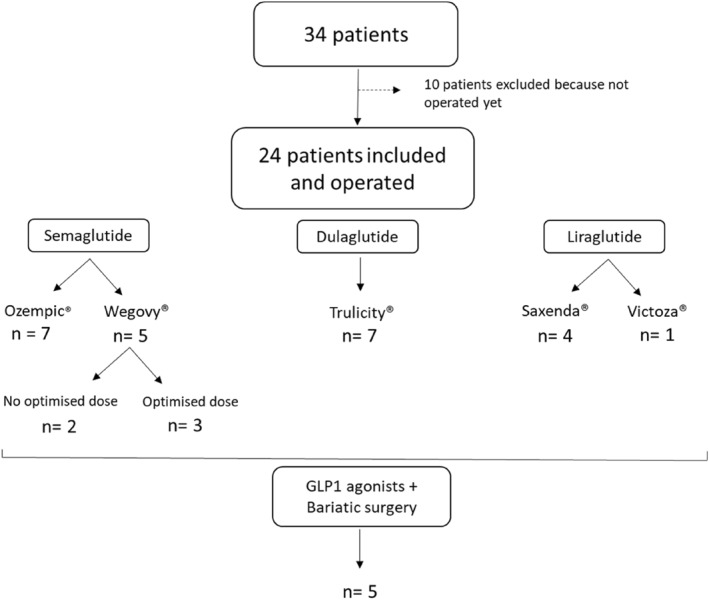
Flowchart.

GLP‐1 agonists were given to 24 patients with obesity in preparation for IHR (Figure 1). The distribution of GLP‐1 agonists was as follows: semaglutide (*n* = 12; 50%), dulaglutide (*n* = 7; 29.2%), and liraglutide (*n* = 5; 20.8%). Among the semaglutide group, there were 5 patients with wegovy, three of whom had the optimized dose of 2.4 mg. Five patients had previous bariatric surgery using a sleeve gastrectomy. In patients who have undergone bariatric surgery, GLP‐1 therapy has been used in cases where bariatric surgery has failed to reduce the weight goal. Treatment with GLP‐1 agonists could be adapted during preparation according to tolerance and weight loss (Table 1). The average duration of treatment was 8.0 ± 3.8 months. There were no serious side effects reported requiring the discontinuation of treatment.

**TABLE 1:** Changes in analog intake during the preoperative optimization.

	Start of the optimization (number of patients)	End of the optimization (number of patients)	Mean treatment length (months ± SD)	Mean dosage
Semaglutide (Ozempic)	6	8	8.88 ± 7.1	0.83 mg/week
Semaglutide (Wegovy)	6	5	11.5 ± 5.8	1.62 mg/week
Dulaglutide (Trulicity)	7	7	11.63 ± 21.7	1.93 mg/week
Liraglutide (Saxenda)	4	4	4 ± 1.4	4.4 mg/week
Liraglutide (Victoza)	1	0	4	1.8 mg/week

There were 52 patients in the control group. Patients were taken from a historical series from January 2019 to June 2021 of consecutive patients who had undergone IHR but who had not benefited from the current prehabilitation recommendations. At the start of patient care, BMIs were comparable between the control and experimental groups, with an overall mean of 40.1 ± 3.6 kg/m^2^ (40.3 ± 2.8 vs. 39.7 ± 12.2, respectively; *p* = 0.26).

### 3.2 Weight Loss by Type of GLP‐1 Agonists

**FIGURE 2:**
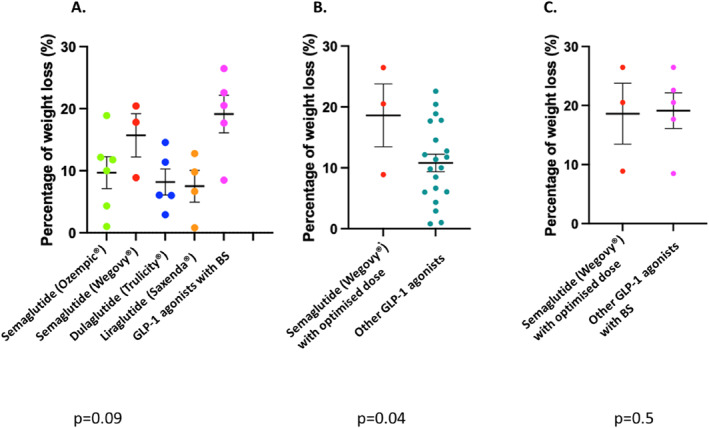
Percentage of weight loss according to the GLP1 agonist type (A); comparison of weight loss between semaglutide (wegovy) with an optimized dose of 2.4 mg and other GLP‐1 agonists (B) and comparison between semaglutide (wegovy) with an optimized dose of 2.4 mg and bariatric surgery (BS) (C).

In the experimental group, the mean initial BMI was 39 ± 12.2 kg/m^2^. The average percentage of weight loss was 11.3 ± 7.4% in the experimental group. Maximum weight loss was observed with semaglutide (Wegovy) compared to other GLP‐1 agonists (Figure 2A). The efficacy of optimized‐dose semaglutide (Wegovy) was significantly superior to other GLP‐1 agonists (*p* = 0.04) (Figure 2B) and optimized‐dose semaglutide (Wegovy) was similar to the efficacy of bariatric surgery (Figure 2C). The average percentage of weight loss was 15.7 ± 6.5% in the semaglutide (Wegovy) group compared to 8.6 ± 5.2% with the other GLP‐1 agonists. Bariatric surgery associated with GLP‐1 agonists had the most efficient effect with 19.1 ± 6.8% weight loss. The success rate of GLP1 agonists was defined as a weight loss that enables the patient to fall within the recommended limits of a BMI ≤ 35 kg/m^2^ before an IHR was reached for 15/24 patients (62.5%).

### 3.3 Surgical Outcomes

After optimization, the BMI in the experimental group was significantly lower than in the control group (34.8 ± 10.5 kg/m^2^ and 40.3 ± 2.8 kg/m^2^,respectively; *p* < 0.001) (Table 2). There were more women in the control group compared to the GLP‐1 agonist group but it was not significant. IH size, VHWG classification, and ASA score were similar between the 2 groups. There was no statistically significant difference in the distribution of other risk factors for postoperative infectious complications, including active tobacco smoking and uncontrolled diabetes mellitus. The mean length of stay was higher in the GLP‐1 agonist group but it was not significant compared to the control group (9 ± 7 days vs. 7.2 ± 7.4 days, respectively; *p* = 0.17). SSI, SSO, and readmission rates were similar. The total complication rate was lower in the GLP‐1 agonist group compared to the control group but it was not significant.

**TABLE 2:** Comparison between the control group without GLP‐1 agonist optimization and the experimental group with GLP‐1 agonists.

	Control group (*n* = 52)	Group GLP‐1 analogs (*n* = 24)	*p*
Mean BMI before GLP‐1	40.3 ± 2.8	39.7 ± 12.2	0.26
Mean BMI after preoperative optimization	40.3 ± 2.8	34.8 ± 10.5	**< 0.001**
Mean age (range)	62.6 ± 10.5	61.4 ± 9.8	0.36
Sex ratio (M/F)	0.33	0.6	0.26
Incisional hernia classification (*n*)			0.48
W1	12	3	
W2	20	9	
W3	20	12	
VHWG classification (*n*)			0.81
1	1	0	
2	35	15	
3	12	6	
4	4	3	
ASA score (*n*)			0.27
1	3	0	
2	24	15	
3	25	9	
Diabete (%)	23 (44.2)	14 (58.3)	0.25
Smoker (%)	1 (1.9)	3 (12.5)	**0.05**
Mean operative time (h)	03:03	3:41	**0.04**
LOS (day mean)	7.2 ± 7.4	9.1 ± 7	0.16
Complication Calvien–Dindo (%)			
≤ II	19 (36.5)	7 (29.1)	0.53
≥ III	12 (23.0)	4 (16.6)	0.52
No complications	21 (35.5)	13 (54.1)	0.26
Surgical site infection (SSI) (%)	12 (23.1)	6 (25.0)	0.85
Surgical site occurrence (SSO) (%)	20 (38.4)	9 (37.5)	0.94
Readmission rate (%)	9 (17.3)	5 (20.1)	0.71

*Note:* Values in bold are significant (*p* < 0.05).

## 4 Discussion

This pilot study presents the outcomes of GLP‐1 agonists preparation before an incisional hernia repair (IHR) in patients with morbid obesity. It showed the efficacy of GLP‐1 agonists in the preoperative management of IHR surgery in order to obtain weight loss (mean %) and the safety of these drugs (no side effects). The results of this study are in line with certain articles in the literature begun to appear demonstrating the value of GLP‐1 agonists in obese patients prior to abdominal wall surgery [20, 21].

Based on actual recommendations in the preoperative optimization, the weight loss must be associated with good glycemic balance (HbA1c < 8%), complete smoking cessation, and respiratory and abdominal muscular training physiotherapy [22]. All patients with a high BMI should be encouraged to lose weight. The optimum BMI for abdominal wall hernia repair remains unclear and no consensus could be achieved on a minimum BMI that patients should be expected to reach before surgery. A weight loss of 7% with medical treatments or a BMI ≤ 35 kg/m^2^ is generally expected before IHR [23, 24]. As it is the case with other types of major surgery, patients of lower weight are likely to have better outcomes. The effect of body composition, particularly sarcopenia, on outcomes is not well understood [25]. In line with European Hernia Society (EHS) guidelines [26], our prehabilitation protocols included in addition to weight loss, smoking cessation, and diabetes control. Nevertheless, some patients in the series were still active smokers, as we considered that the risk of strangulation of the hernia warranted surgery without delay.

As obesity increases the risk of recurrence, impaired wound healing, and local infection [27], it is currently recommended to not perform an elective IHR for patients with a BMI ≥ 50 kg/m^2^ [28]. For this purpose, every surgeon must suggest that patients lose weight, and in order to help patients to achieve this goal, several strategies have been proposed. Spontaneous weight loss through dieting alone, without supervision, is often a failure. Ssentongo et al. showed that this strategy was associated with more than 80% failure (60% had a stable weight, 20% lost more than one point of BMI, and 20% gained weight before surgery) [29]. Liang et al. studied nutritional prehabilitation versus standard counseling before hernia surgery in a randomized study [28]. Among the 118 randomized patients [28], prehabilitation was associated with a higher percentage of patients who lost weight and achieved weight loss goals. This strategy permitted an increase in the access rate to parietal surgery (82% vs. 57%), but only 27% of the nutrition group achieved their weight loss objective of more than 7%. Prehabilitation was also associated with a higher dropout rate (7% of the patients left the trial) and need for emergent repair. In our study, the weight loss objective was achieved in 62.5% of cases with GLP‐1 agonist optimization; the average percentage of weight loss was 11.3 ± 7.4% in this group and 15.7 ± 6.5% in the Wegovy group.

Bariatric surgery has recently been validated by the French health authorities for patients with severe obesity and IH as a comorbidity [5]. Thus, bariatric surgery can be an intermediate step in optimizing patients with severe obesity for IHR [13]. However, medical treatments, such as GLP‐1 agonists (as weekly semaglutide), seem also to be interesting to achieve significant weight loss in patients with severe obesity and comorbidities [7]. Ozempic and Wegovy contain the same molecule (semaglutide) but in different dosages and for different indications. The indication for Ozempic is for control of diabetes, whereas the indication for Wegovy is for weight loss. For Ozempic, the doses range from 0.25 to 1 mg per week, whereas from 0.25 to 2.5 mg per week for Wegovy. The superiority of Wegovy may indeed lie in the fact that there is a dose–response relationship with weight loss [30]. New GLP‐1 agonists will soon be available on the market with improved efficacy [31].

The advantage of GLP1 agonists is that, unlike bariatric surgery, they are noninvasive. They are also often patients who have undergone multiple operations, and bariatric surgery can be difficult because of the risk of adhesions. However, long‐term treatment with GLP‐1 agonists comes at a significant cost to society, especially since it must be continued over a long period of time and its cessation leads to weight regain. The article by Docimo S et al. [32] compared the cost‐effectiveness of GLP‐1 agonists and bariatric surgery. The authors suggest that bariatric surgery seems to be cost saving compared with GLP‐1 agonists in the treatment of class II obesity. The high cost of ongoing use of GLP‐1 agonists, such as Saxenda and Wegovy, surpasses the cost of Roux‐en‐Y gastric bypass in less than a year and sleeve gastrectomy within 9 months.

This study has several limitations. The number of subjects included is small and lacks statistical power to assess the impact of treatment on postoperative complications and length of stay. Differences in BMI collection methods between the control and experimental group and the fact that some patients also had bariatric surgery could be a potential bias. The overrepresentation of women in the control group may be also a source of bias. Although the rate of overall complications was higher in the control group than in the GLP‐1 agonists group, this difference did not reach significance and needs to be confirmed in larger cohorts. Furthermore, the types of GLP‐1 agonists are not homogeneous and the doses vary. Too short of time period between starting the medication and surgery was an exclusion criteria. The regular implementation of preoptimization with GLP‐1 agonists could be limited because the drug is no longer reimbursed due to the massive theoretical budgetary consequences for healthcare systems (around 250 euros/month) in France. Very few studies in the literature have examined the value of this type of treatment in the preoperative optimization of patients with obesity prior to surgery.

## 5 Conclusion

This study demonstrated the safety and efficacy of GLP‐1 agonists in the preoperative weight loss required before IHR surgery in patients with severe obesity. Although this study did not show a significant reduction in the number of postoperative complications or in the length of stay, more than half of the patients managed to achieve a BMI below 35 kg/m^2^ before surgery. Further studies on a larger number of patients are needed to investigate the impact of GLP‐1 agonists before complex abdominal wall repair.

## Author Contributions

**Benoit Romain:** conceptualization, formal analysis, methodology, project administration, supervision, validation, visualization, writing – original draft, writing – review & editing. **Vincent Pfirsch:** data curation. **Simone Manfredelli:** formal analysis, methodology. **Thomas Leroi:** writing – review & editing. **Fadi Salman:** data curation. **Ouidad Sami:** investigation, resources. **Diane Westerfeld‐Ruillier:** validation. **Séverine Ledoux:** validation. **David Moszkowicz:** validation, writing – original draft, writing – review & editing.

## Ethics Statement

The study was approved by the national research ethics committee.

## Consent

Informed consent was obtained from all individual participants included in the study.

## Conflicts of Interest

The authors declare no conflicts of interest.

## Data Availability

The data are available on request from the authors.
